# Relative clause reading in hearing impairment: different profiles of syntactic impairment

**DOI:** 10.3389/fpsyg.2014.01229

**Published:** 2014-11-07

**Authors:** Ronit Szterman, Naama Friedmann

**Affiliations:** Language and Brain Lab, Sagol School of Neuroscience and School of Education, Tel Aviv UniversityTel Aviv, Israel

**Keywords:** hearing impairment, Hebrew, movement, reading, relative clauses, syntax, syntactic impairment, syntactic tree

## Abstract

Children with hearing impairment show difficulties in sentences derived by Wh-movement, such as relative clauses and Wh-questions. This study examines the nature of this deficit in 48 hearing impaired children aged 9–12 years and 38 hearing controls. The task involved reading aloud and paraphrasing of object relatives that include a noun-verb heterophonic homograph. The correct pronunciation of the homograph in these sentences depended upon the correct construction of the syntactic structure of the sentence. An analysis of the reading and paraphrasing of each participant exposed two different patterns of syntactic impairment. Some hearing-impaired children paraphrased the object relatives incorrectly but could still read the homograph, indicating impaired assignment of thematic roles alongside good syntactic structure building; other hearing-impaired children could neither read the homograph nor paraphrase the sentence, indicating a structural deficit in the syntactic tree. Further testing of these children confirmed the different impairments: some are impaired only in Wh-movement, whereas others have CP impairment. The syntactic impairment correlated with whether or not a hearing device was fitted by the age of 1 year, but not with the type of hearing device or the depth of hearing loss: children who had a hearing device fitted during the first year of life had better syntactic abilities than children whose hearing devices were fitted later.

## Introduction

Children with hearing impairment encounter difficulties in understanding non-canonical sentences that are derived by movement of phrases (Berent, 1988, 1996a,b; De Villiers et al., 1994; Friedmann and Szterman, 2006, 2011; Friedmann et al., 2010). This deficit probably stems from limited language input during the critical period for the acquisition of the syntax of a first language (Yoshinaga-Itano and Apuzzo, 1998a,b; Mayberry et al., 2002, 2011; Yoshinaga-Itano, 2003; Friedmann and Szterman, 2006).

The aim of the current study was to learn about the nature of the syntactic deficit of children with hearing impairment. To do so, we used a novel task that allowed us to evaluate various sources for the syntactic difficulty. The task also allowed us to examine whether they experience comprehension difficulties also when the sentences are written and presented for an unlimited time, and provided a window to the reading comprehension difficulties often reported for hearing impaired children.

Studies that assessed the syntactic abilities of English-, Hebrew-, Palestinian Arabic-, and Italian-speaking hearing impaired children found difficulties in the comprehension and production of object relative clauses (English: Quigley et al., 1974a; Berent, 1988; De Villiers, 1988, Hebrew: Szterman and Friedmann, 2003, 2007; Friedmann and Szterman, 2006, 2011; Friedmann et al., 2010, Arabic: Haddad-Hanna and Friedmann, 2009; Friedmann et al., 2010; Friedmann and Haddad-Hanna, 2014; and Italian: Volpato and Adani, 2009), in the comprehension and production of object questions (English: Quigley et al., 1974b; Berent, 1996b, Hebrew: Nave et al., 2009; Friedmann and Szterman, 2011; Szterman and Friedmann, 2014, Standard Arabic and Palestinian Arabic: Friedmann et al., 2010; Haddad-Hanna and Friedmann, 2014), and in the comprehension of topicalization structures (Hebrew: Friedmann and Szterman, 2006, Arabic: Haddad-Hanna and Friedmann, 2009; Friedmann and Haddad-Hanna, 2014).

A look at these three impaired structures: object relative clauses, object questions, and topicalization structures suggests a common syntactic characteristic: they are all derived by movement of a phrase that results in a non-canonical order of the arguments in the sentence, as shown in examples (1)–(3) (movement is depicted in these examples by arrows).

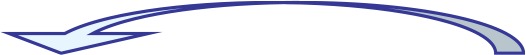
  (1) Object relative: This is the girl_1_ that the grandma drew t_1_.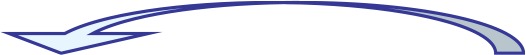  (2) Object question: Which girl_1_ did the grandma draw t_1_?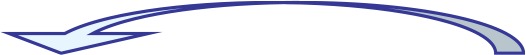  (3) Topicalization: This girl_1_, the grandma drew t_1_.

In every sentence, the verb identifies the roles of the participants in the event, and assigns thematic roles to its arguments. In sentences 1–3, the verb *drew* assigns a thematic role of an Agent—the person who draws, and a Theme—the object that was drawn. In Hebrew, as in English, verbs usually assign the theme role to the noun phrase (NP) that follows them. However, in sentences like 1–3, the Theme precedes, rather than follows, the verb. Linguistic theory suggests that such sentences are derived by syntactic movement to a position that is hierarchically higher (which typically appears earlier in the sentence) (Chomsky, 1981, 1986, 1995; Rizzi, 1990; this operation is termed “internal merge” in more recent frameworks, see Chomsky, 2000, 2001). The moved theme leaves a trace [marked in sentences (1)–(4) by t_1_] (or a copy according to more recent linguistic frameworks) in its original position. The verb assigns the thematic role to the object position, and the moved object is linked to its trace with a “chain” of movement. Thus, to understand a sentence with syntactic movement, two operations are required: constructing a syntactic tree that includes a trace at the position from which the element has moved, and creating the chain between the trace and the moved element, to allow for the comprehension of the roles of the participants in the sentence.

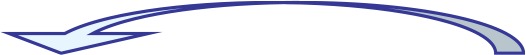
  (4) The girl_1_ that the grandma drew t_1_ is very kind.

For example, sentence (4) includes an object relative clause. In object relatives, the object of the relative clause (in this case, *the girl)* moves to a position earlier in the sentence^1^. When the object *the girl* moves, it leaves behind a trace in the embedded object position. Thus, to correctly understand such a sentence, the appropriate syntactic structure of the sentence should be constructed. This structure should include the moved element in the correct syntactic position, the relativizer (embedding marker), and an empty element, a trace of movement, should be placed in the correct position. This is not enough, though. In order to understand the role of the moved element, the chain should be established, namely, the link between the original position and the moved argument (illustrated by the arrow in 4).

A step-by-step description of the parsing the hearer needs to perform in order to understand who did what to whom in an object relative like (4) would be the following: upon hearing the NP *the girl*, the hearer is waiting for a thematic role for this NP. Once the word *that* is heard, the NP needs to be stored in a syntactic STM store until it can receive its role, and the search for a gap, the position from which this NP has moved, begins. When the subject NP *the grandma* arrives, it is also put into the syntactic store, until the verb finally arrives. When the verb arrives, the hearer accesses its entry in the syntactic lexicon together with the thematic roles it assigns. Then, the subject receives the thematic role of the Agent, the gap (trace) is postulated, and the moved element is re-accessed at this point. In processing terms, this is where the chain is constructed, between the moved element and the position in which it originated. One may think of this stage in processing terms as the re-activation of the correct antecedent at the gap (Nicol and Swinney, 1989). Impaired comprehension of a sentence with movement can result from a deficit in either of these operations.

In the current study we tried to determine which of these operations is responsible for the difficulty hearing impaired children have with object relatives. We made a distinction between the steps that require constructing the syntactic structure of the object relative clause, including the assumption of a trace, and operations related to the identification of the thematic role of the moved element (the reactivation of the appropriate NP in the trace position and the assignment of the Theme role to it).

We used a task that allowed us to separately evaluate structure building and thematic role assignment to a moved NP. This task was already used to identify the source of the deficit in the comprehension of movement-derived sentences in children with syntactic SLI and in individuals with agrammatic aphasia (Friedmann et al., 2006; Friedmann and Novogrodsky, 2007). This task used the fact that the correct pronunciation of noun-verb heterophonic homographs (i.e., words that are written the same but sound differently, like *dove*) in oral reading requires the analysis of the syntactic position of the homograph. For example, in sentence (5), the word *dove* appears as the object, and is therefore read as a noun (/dᴧv/), whereas in sentence (6) it appears as the main verb, and therefore read as a verb (/do℧v/).

(5) We saw a dove flying in the sky.
(6) The dolphin dove into the river.

The dependency between correct reading aloud and the construction of the syntactic structure of the sentence served us to evaluate the way children with hearing impairment process relative clauses. We asked the participants to read aloud object relatives in which a noun-verb heterophonic homographs appeared immediately after the trace position. Thus, to read the homograph correctly in these sentences, the reader would have to be able to construct a trace after the verb, at the object position. For example, to read correctly the homograph *presents* in sentence (7), the reader has to know that the object of *received* is the trace of *the chart*, and therefore *presents* cannot be the object of *received*, and is rather the main verb. However, if the trace is not identified, the embedded verb *received* might be missing an object, so the homograph might be read as a noun, the object of *received*.

(7) The column chart_1_ [that the scientist received t_1_] presents the reading of the two groups.

Hebrew orthography allows for many degrees of freedom in the conversion of graphemes to phonemes: not all the vowels are represented in writing, some consonant letters are phonologically ambiguous, and the stress position is not marked (Friedmann and Lukov, 2008). This creates many heterophonic homographs, and for many of them one reading is a noun and the other is a verb. Many of these homographs can be used in a study of children's comprehension, because both their meanings are well-known to children.

The word MXBRT 
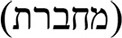
, for example, can be read, because of the underrepresentation of vowels in Hebrew, either as a noun, /maxberet/, notebook, or as a verb, /mexaberet/, creates-feminine-3rd person-singular. Example (8) shows a sentence we used, in which the reader needs to parse the sentence and identify the syntactic role of this homograph in order to read it in a way that is appropriate for the sentence. In (8), the homograph MXBRT functions as the main verb, and is located immediately after the trace position.

(8) Ha-ganenet she-ha-yalda ohevet t_1_ MXBRT sipurim.
  *The-kindergarten-teacher*_1_*that-the-girl loves t*_1_
  *writes/a-notebook-of stories*^2^.
(9) Correct reading:
  The kindergarten teacher who the girl loves writes stories.
(10) Incorrect reading:
  The kindergarten teacher who the girl loves a notebook-of stories.

The rationale behind this task is that if the reader postulates a trace immediately after the verb, he should know that the trace is the complement of the embedded verb *loves*. Therefore, he would analyze the homograph as the main verb, resulting with a correct reading of the homograph, as a verb (example 9). However, if the reader cannot construct a trace at the required position, the embedded verb *loves* would appear to be lacking a complement. Because the reader knows the argument structure requirements of the verb *loves*, which requires a Theme as a complement, he will search for a theme. This might lead to an incorrect reading of the homograph as the complement of the embedded verb. In this case, the written sentence (8) will be read incorrectly as in (10) *loves a notebook of stories*, where the homograph would be read as a noun. The ungrammaticality of such a reading results from the fact that the verb *loves* can only assign one thematic role of a Theme, and if the reader takes the NP after the verb to be its object and receive a Theme role, the moved element remains role-less.

The crucial point here is that even the assumption of an empty category at the correct structural position, which is enough for the correct reading aloud of the homograph, does not guarantee the correct interpretation of the sentence. If the assignment of thematic roles to the displaced NP is impaired because of a failure to establish the chain between it and its original position, the interpretation of the sentence might still be flawed. For example, an inability to assign the thematic role to the moved NP in sentence (9) might result in understanding the sentence with reversed roles, as if the kindergarten teacher loves the girl. In processing terms (see for example Nicol and Swinney, 1989; Zurif et al., 1993), this might be a result of the activation of an incorrect NP at the gap position, or not knowing which of the NPs to re-activate. Such difficulties in assignment of thematic roles can be identified by asking the reader to paraphrase the sentence.

Thus, oral reading of the homograph placed immediately after the trace position can serve as a sensitive indicator for the construction of the syntactic position of the moved element and the postulation of an empty category in its original position. The paraphrasing of the sentence can serve as an indicator for whether or not the thematic roles were correctly assigned to the moved element (and the rest of the NPs in the sentence).

If the difficulties in the comprehension of object relatives in hearing impaired children result from the inability to construct the syntactic structure and the trace, poor performance in the reading task is expected, with a tendency to read the homographic verb as the object noun. If, however, the difficulties are a result of a deficit in thematic role assignment to moved elements, with unimpaired trace identification and with good structure-building, correct reading of the homograph is expected, accompanied with difficulties in the assignment of thematic roles in the paraphrasing task. Thus, the assessment of the performance of hearing impaired children in reading and in paraphrasing of such sentences can shed light on the source of their impairment in sentences with syntactic movement.

The task can also shed light on a further open issue in the study of hearing impaired children: it is often mentioned that hearing impaired children have considerable difficulties in reading comprehension (Trybus and Karchmer, 1977; Moog and Geers, 1985; Allen, 1986; Musselman, 2000; Traxler, 2000; Moeller et al., 2006; Luckner and Handley, 2008). It might be that their reading comprehension difficulty is actually unrelated to reading, but rather stems from their syntactic difficulties. The pattern of these children's reading and comprehension of the written relative clauses (in comparison with simple sentences) might give us a further hint as to this issue.

## Method

### Participants

The participants were 48 Hebrew-speaking children with hearing impairment. They were 27 boys and 21 girls, aged 9;1 and 12;6 years (*M* = 10;7, *SD* = 0;10). They had moderate to severe hearing loss and were trained in oral language. At the time of testing, they were studying in primary schools in hearing classes with inclusive schooling using oral education, and each of them received additional support from a special teacher of the deaf, 2–4 h a week. All the participants consistently wore binaural hearing aids (23 children) or used cochlear implants (25 children, one of them in combination with a hearing aid on the other ear), and they all passed a hearing screening test that they performed while wearing their hearing aids/ implants, in which they were asked to repeat 10 sentences that included sibilants and were read to them by the experimenter with her lips concealed. Forty six of the participants had hearing loss from birth (based on early detection or genetic source of the hearing loss) and two had probable progressive hearing loss.

The background information on the participants' hearing is presented on Table 1. Subject files included no other disabilities, and in all cases neither parent was deaf, and they all came from a family that spoke only Hebrew. An informed consent statement approved by the Ministry of Education Review Board was signed by all participants' parents.

**Table 1:** **Background information on the hearing impaired participants**.

**no**.	**Participant**	**Age**	**Gender**	**Age at diagnosis**	**Age at the beginning of intervention (hearing aids fitted)**	**Type of hearing loss**	**Etiology**	**Hearing loss dB (right and left)^a^**	**Device (CI = cochlear implant, HA = 2 hearing aids)**	**Age at first implantation**
1	DOH	10;10	Male	0;6	1;0	Sensorineural	Unknown	r-90, l-70	HA	
2	DOD	11;5	Female	2;6	3;6	Sensorineural	Unknown	r-60, l-65	HA	
3	CEB	9;7	Female	0;0	0;2	Sensorineural	Genetic	r-65, l-70	HA	
4	TBN	9;8	Male	0;0	0;6	Sensorineural	Genetic	r-50, l-50	HA	
5	SIG	10;6	Female	3;0	7;0	Sensorineural	Unknown	r-85, l-75	HA	
6	SAV	11;11	Male	0;6	3;0	Sensorineural	Unknown	r-45, l-50	HA	
7	AVC	10;4	Male	0;0		Sensorineural	Genetic	r-85, l-85	HA	
8	IVL	9;8	Male	0;0	3;6	Combined	Middle ear deformation	r-50, l-50	HA	
9	ORC	9;9	Male	3;0	7;0	Sensorineural	Unknown	r-65, l-120	HA	
10	TOS	10;10	Male	1;4	2;6	Combined	Genetic	r-80, l-80	HA	
11	NEA	10;3	Male	3;0	3;0	Sensorineural	Unknown	r-65, l-65	HA	
12	KEM	11;1	Female	0;6	3;0	Sensorineural	Genetic	r-70, l-75	HA	
13	ROS	10;0	Male	0;0	0;9	Sensorineural	Genetic	r-55, l-55	HA	
14	TAM	9;8	Male	0;3	0;6	Sensorineural	Preterm	r-50, l-55	HA	
15	YEO	12;0	Male	5;0		Combined	Unknown	r-50, l-55	HA	
16	DAC	10;1	Male	3;0	3;0	Combined	Unknown	r-60, l-65	HA	
17	YAO	10;1	Female	3;0	3;0	Sensorineural	Genetic	r-60, l-65	HA	
18	YIL	10;6	Female	5;0	5;0	Sensorineural	Genetic	r-80, l-80	HA	
19	LIS	11;7	Female			Sensorineural	Unknown	r-70, l-70	HA	
20	ROP	10;9	Male	0;3	1;0	Sensorineural	Genetic	r-50, l-50	HA	
21	DAM	10;1	Female	0;10	1;0	Sensorineural	Genetic	r-65, l-65	HA	
22	OFC	9;5	Male	2;0	4;0	Combined	Unknown	r-80, l-75	HA	
23	TOH	10;0	Male	0;9	0;11	Sensorineural	Unknown	r-115, l-95	HA	
24	HIM	9;11	Female	0;7	0;8	Sensorineural	Unknown		CI	1;7
25	TAC	11;3	Female	0;6	0;10	Sensorineural	Syndrome		CI	2;2
26	YOD	10;3	Male	0;6		Sensorineural	Unknown		2 CI	1;5
27	NAH	10;6	Male	0;0	0;2	Sensorineural	Syndrome		2 CI	1;0
28	SAS	10;6	Female	0;0	1;0	Sensorineural	Unknown		CI+HI	2;2
29	RON	10;9	Female	0;2	0;3	Sensorineural	Genetic		CI	1;0
30	YIB	10;3	Male	0;9	1;2	Sensorineural	Unknown		CI	1;6
31	EDY	9;6	Female	1;6	1;9	Sensorineural	Genetic		CI	4;5
32	LIH	9;1	Female	0;2	0;3	Sensorineural	Unknown		CI	1;0
33	LER	9;11	Male	0;0		Sensorineural	Genetic		2 CI	1;3
34	RAR	11;7	Male	0;0	1;0	Sensorineural	Unknown		CI	5;0
35	LIW	10;1	Male	0;6		Sensorineural	Genetic		CI	1;0
36	LIA	10;0	Female	0;8		Sensorineural	Unknown		2 CI	1;3
37	KOZ	11;8	Male	0;3	0;3	Sensorineural	Genetic		CI	1;0
38	AIR	11;5	Female	1;0	1;0	Sensorineural	Genetic		2 CI	1;8
39	LIC	11;3	Female	0;8	0;8	Sensorineural	Genetic		2 CI	1;2
40	ZIZ	11;0	Male	0;9	0;9	Sensorineural	Unknown		CI	2;1
41	ORS	9;10	Male	0;11	1;0	Sensorineural	Genetic		CI	2;1
42	MAK	11;1	Male	0;9	0;9	Sensorineural	Unknown		CI	4;0
43	TOS	12;6	Male	0;10	1;0	Sensorineural	Unknown		CI	4;9
44	ODC	10;10	Female	0;0	0;3	Sensorineural	Genetic		CI	3;0
45	CBN	12;2	Male	0;0	0;6	Sensorineural	Genetic		CI	3;6
46	LIL	10;10	Female	0;0	0;4	Sensorineural	Genetic		2 CI	5;0
47	MAL	10;10	Female	0;0	0;4	Sensorineural	Genetic		2 CI	2;6
48	YIC	11;5	Female	0;0	0;6	Sensorineural	Genetic		CI	1;6

a *All the participants with unilateral cochlear implant had a hearing loss of 95–105 dB in the unaided ear (pure tone average of 500, 1000, and 2000 Hz)*.

*r, right ear; l, left ear; CI, unilateral cochlear implant; 2CI, bilateral cochlear implants*.

To evaluate oral reading at the single word level, and exclude participants with severe dyslexia, we tested 43 of the hearing-impaired participants using the TILTAN screening test (Friedmann and Gvion, 2003), which was developed to identify subtypes of dyslexia. The screening test includes oral reading of 136 single words, 30 word pairs, and 40 non-words. The test includes words of various types that can reveal the different types of dyslexia. The results of the screening test indicated that two girls of the initial group of 50 participants, had a significant deficit in reading words, and therefore they were excluded from this study.

#### Control group

The participants in the control group were 38 typically-developing hearing Hebrew-speaking children in fourth grade (mean age = 9;8, *SD* = 0;5). They met the criteria of normal hearing, normal language development, and had no reports of neurological development difficulties or socio-emotional problems. They were taken from public schools serving a middle-class population, similarly to the participants with hearing loss.

## Materials

The test included 20 sentences in which the main verbs that were heterophonic-homographs of nouns. Half of the sentences were relative clauses, and half were simple control sentences. The relative clauses were center-embedded object relatives with the relativizer “she-”, which is obligatory in Hebrew relative clauses (it is also used as the embedding marker for sentential complements). The homographic verbs appeared in the relative clauses immediately after the trace. Because we needed to add the homograph after the trace position, we used center-embedded object relatives. Hebrew-speaking children at the ages tested already understand such center embedded relatives, as shown in Friedmann and Novogrodsky (2007) and in the performance of the hearing control participants of the current study reported below.

Example (11) shows an object relative clause with the homograph 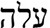, which can be read either as the verb /ala/, meaning ascended, climbed, or as the noun /ale/, a leaf. For each sentence with a relative clause, a control sentence that included the same homograph was constructed that was a length-matched simple sentence without movement (12).

(11) ha-madrix_1_ she-ha-yeled ra'a t_1_ ala al ha-har.
  *The-guide*_1_ *that-the-boy saw t*_1_ *climbed the mountain*
  The guide that the boy saw climbed the mountain.
(12) ha-sus im ha-zanav ha-gadol ala al ha-deshe.
  The-horse with the-tail the-big climbed on the grass
  The horse with the big tail stepped on the grass.

The relative clauses were constructed so that the homograph in its incorrect, noun reading would be a semantically and syntactically appropriate complement of the embedded verb. For this aim we chose embedded verbs that could take the noun homographs as their object. There were no morphological cues that could identify the homograph as a verb or a noun. The fact that the homograph was not preceded by an article could also not be used as a cue for it being a verb, because in Hebrew indefinite nouns appear without any determiner. To prevent reliance on semantics and world knowledge cues in the interpretation of the sentences, the relative clauses were semantically reversible, namely, the two NPs in the sentence could semantically both serve as the agent and as the theme of the embedded verb, and both could serve as the agent of the main verb. For example, in (11) it is possible both for the guide to see the boy and for the boy to see the guide, and both the boy and the guide can climb the mountain, and therefore comprehension cannot be based solely on the semantics of the lexical items, and has to rely on syntax. The sentences included only homographs for which the verb and the noun meanings were different enough to permit reliable judgment of which meaning was selected in the speakers' paraphrases (like *dove, tear, presents*, and *objects* in English). The homographs were simple and frequent words that school-age children are acquainted both with their verb and with their noun meaning.

The homographs in the sentences were either biased toward the incorrect (noun) meaning or had two meanings with similar frequency. The dominant meaning was determined by Friedmann and Novogrodsky (2007) according to judgments of 50 Hebrew-speaking adults and 50 Hebrew-speaking children without language impairment. The 50 adults (aged 18–55) were asked to determine for each heterophonic homograph which of the meanings is more frequent—they could either circle one meaning or say that they had similar frequency. According to their judgments we classified one homograph as biased toward the noun reading, and the rest as equi-biased (according to Onifer and Swinney's 1981 criterion for primary meaning, of a meaning preferred by at least 75% of the judges)^3^. In addition, Friedmann and Novogrodsky (2007) presented a list of the homographs as single words to 50 children in 4th–7th grade and asked them to read them aloud, and noted how often each homograph was read as a noun or as a verb. The results were similar to the results of the adults, and even more strongly biased toward the noun reading. Eight of the homographs were strongly biased toward the noun reading (more than 75% of the children read them as nouns), and two homographs were biased toward the noun but less strongly (69 and 74% of the children read them as nouns).

The test sentences were divided into two blocks; one block was administered in each of two sessions, in each block each homograph appeared only once. Each block included five relative clauses and five control sentences, in random order. The second block included the control sentences for the five target sentences in the first block, and five relative clauses whose control sentences appeared in the first block.

### Procedure

The sentences were printed on a white page, presented in front of the participants. We asked the participants to read each sentence aloud, and then to explain it in their own words. To explain what “in your own words” mean, we started with a simple sentence, and gave the participants feedback on their paraphrase. For the rest of the task we did not give any feedback as to their success or failure to understand the sentences, only commented on whether or not their paraphrase was complete, and gave general encouragement.

If, in the paraphrase, the child only explained part of the sentence or if the paraphrase was not sufficiently clear to determine the thematic roles the participant assigned to the NPs in the sentence, we asked a clarification question. For example, if a participant said in the paraphrase of sentence (11) “The boy saw the guide”, and ignored the main verb, we asked “and what else happened in the sentence?”, and when a participant said “The guide climbed the mountain”, we asked “and what about the boy?”. When the participants repeated the written sentence, we asked them to try again, and explain the sentence in their own words.

No time limit was set. The sentences remained in front of the participants throughout the reading and paraphrasing task, to reduce demands on memory. The two 10-sentence blocks (in which the same homographs were incorporated in different sentences) were administered 1 or 2 weeks apart.

### Analyses

Reading aloud was classified as correct if the homograph was read correctly, immediately or after self-correction. Paraphrases were classified as correct if they described correctly the thematic roles of the two NPs in the sentence and the arguments of the two verbs—the main verb and the embedded verb. Paraphrases in which one or more thematic roles were incorrect were counted as incorrect.

## Results at the group level

**Figure 1:**
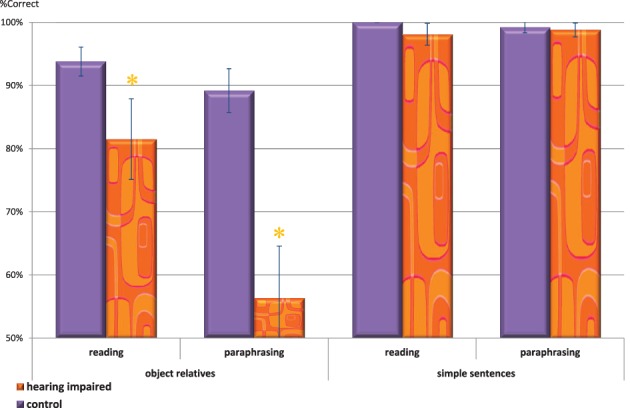
**Reading and paraphrasing of relative clauses and simple control sentences in the two groups**. Error bars present 95% confidence interval. ^*^p < 0.001 in the comparison between the groups.

The results, summarized in Figure 1, indicate that the hearing impaired children have a severe difficulty in object relative clauses. The group's reading and paraphrasing of the object relatives were significantly poorer than that of the control group. Importantly, the difficulty exhibited by the hearing impaired group did not result from a general difficulty in reading or in paraphrasing. They performed very well in reading and paraphrasing the simple length-matched control sentences, which did not include movement, and their performance was virtually like that of the control participants in these sentences.

### Reading aloud

As a group, the hearing impaired children showed difficulty in the reading of the homograph in the object relatives, and their performance (*M* = 81.5%, *SD* = 22.6%) was significantly poorer than that of the participants in the control group (*M* = 93.8%, *SD* = 7.2%), Wald chi = 25.41, *p* < 0.001 (analyzed using a mixed logit model, with by-participant and by-item random effects).

Out of the 480 homographs in object relatives that the group of hearing impaired children read, only 391 were read correctly. All the 89 sentences that were read erroneously included incorrect reading of the homographic verb as a noun [see (13) for an example for the way they read the sentence with the homograph 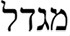, MGDL, which can be read either as a verb /megadeal/ “grows”, or as the noun /migdal/ “tower”].

Other important information can be gained by looking at the children's reading of other parts of the relative clause. Some of the children canceled the subordination in the sentence by changing the relativizer “she” into the word “shel”, *of* in Hebrew (14), or into a coordination marker.

(13)
  - Target sentence:
    ha-baxur_1_ she-aba cilem t_1_ megadel taltalim arukim.
    the-guy_1_ that-dad photographed t_1_ grows curls long
    The guy that dad photographed grows long curls.
  - Reading the homograph verb as a noun:
    ha-baxur she-aba cilem migdal taltalim arukim.
    the-guy that-dad photographed tower curls long
    The guy that dad photographed a tower of ^4^ long curls.
(14) Canceling the subordination:
  ha-baxur shel aba cilem megadel taltalim arukim.
  the-guy of dad photographed grows curls long
  Dad's guy photographed grows long curls.

Importantly, the marked difficulty in reading the homograph cannot be ascribed to a reading impairment, but rather stems from the syntactic structure of the relative clause: When the same homographs were incorporated in simple sentences, the hearing impaired participants read them very well (98% correct), and significantly better than when they were incorporated in object relatives (81%), Wald chi = 48.32, *p* < 0.001 ^5^.

### Paraphrasing

The paraphrasing task also indicated that as a group, the hearing impaired children have a considerable difficulty in paraphrasing the object relatives. Their performance in paraphrasing of the object relatives (*M* = 56.9%, *SD* = 29.4%) was significantly poorer than that of the participants in the control group (*M* = 89.2%, *SD* = 10.9%), Wald chi = 95.47, *p* < 0.001. This difficulty did not result from a general problem in the task of paraphrasing, as indicated by their good paraphrasing of the control sentences (*M* = 98.8%, *SD* = 3.9%), which was significantly better than their paraphrasing of the object relatives, Wald chi = 67.49, *p* < 0.001, and not differently from that of the controls, Wald chi = 0.08, *p* = 0.77.

Out of 480 object relatives the hearing impaired children paraphrased, only 270 sentences (56%) were paraphrased correctly. In marked contrast, when they paraphrased the simple control sentences, they did it well, and made errors only on 6 sentences out of 480 sentences (1%).

There was a main effect for sentence type, Wald chi = 67.49, *p* < 0.001, no significant main effect of group, but importantly, a significant interaction between sentence type and group, Wald chi = 6.06, *p* = 0.01.

We further analyzed the paraphrasing errors. The detailed distribution of the paraphrasing errors in the hearing impaired group is presented in Table 2. One of the two most common types of paraphrasing errors was incorrect thematic role assignment. The incorrect thematic role assignment errors, which accounted for 55% of the errors in paraphrasing, included three types of incorrect thematic role assignment. One was ascribing the predicate of the main clause to the subject of the relative clause, which occurred in 26% of the thematic role errors [see example (15a) for a paraphrase that one of the participants gave for sentence (15)]. Another error type in thematic role assignment involved ascribing the predicate of the relative clause to the subject of the main clause, which occurred in 33% of the thematic role errors (15b). Additional 41% of the thematic role errors in paraphrasing involved both ascribing the predicate of the main clause to the subject of the relative clause, and ascribing the predicate of the relative clause to the subject of the main clause (15c).

**Table 2:** **The distribution of the paraphrasing errors of the center-embedded object relatives in the hearing impaired group**.

**Paraphrasing error type**	**% of paraphrasing errors**
Incorrect thematic role assignment	55.0
Treating the homograph as a noun	32.0
Cancelation of the subordination	7.5
“I don't understand” responses	5.5

Another frequent type of error involved the interpretation of the homograph as a noun (16). In these paraphrases the hearing impaired children tried to make sense of the sentences somehow and to reach an interpretation in which all NPs in the sentence receive a role. Additional incorrect responses included cancelation of the subordination and “I don't understand” responses. Some responses included more than one type of error.

(15) Target sentence
  ha-baxur_1_ she-aba cilem t_1_ megadel taltalim arukim.
  *the-guy*_1_ *that-dad photographed t*_1_ *grows curls long*
  The guy that dad photographed grows long curls.
  Correct paraphrasing:
  Aba cilem et ha-baxur ve-ha-baxur megadel taltlim arukim.
  Dad photographed ACC the-guy and the guy grows long curls
  Dad photographed the guy and the guy grows long curls.
  Incorrect thematic role assignment
  - **Ascribing the predicate of the main clause to the subject of the relative clause**
    Aba, yesh lo… hu megadeal taltalim arukim.
    Daddy, there's to-him… he grows curls long
    Daddy, he has… he grows long curls.
  - **Ascribing the predicate of the relative clause to the subject of the main clause**
    Ha-baxur she-cilem et aba hu megadeal taltalim.
    The-guy that-photographed ACC daddy he grows curls
    The guy that photographed daddy, he grows curls.
  - **Both ascribing the predicate of the main clause to the subject of the relative clause, and ascribing the predicate of the relative clause to the subject of the main clause**
    Aba megadel taltalim ve-ha-baxur cilem oto
    daddy grows curls and-the-guy photographed him
    Daddy grows curls and-the-guy photographed him
(16) **Treating the homograph as a noun**
  Aba cilem et ha-migdal taltalim arukim.
  Daddy photographed ACC the-tower curls long
  Daddy photographed the long curls tower.

In 121 of the sentences, the hearing impaired children paraphrased the sentences erroneously although their reading was correct. In 89 other cases the incorrect paraphrasing of the center-embedded object relatives was a result of incorrect reading of the verb-noun homograph.

## Results at the individual level: crucially different profiles

### Group-level analysis hides two different profiles of impairment

The analysis of the performance at the group level shows a significant difficulty both in reading homographs placed after the trace position, and in paraphrasing object relatives. However, the group analysis hides crucially different profiles within the hearing impaired group. When we analyzed the performance of each of the hearing impaired participants in reading and paraphrasing, we found that they do not all show the same pattern. In fact, three different patterns could be detected. One subgroup of hearing impaired children read the homographs in the object relative clauses correctly, much like the controls, but failed to explain the meaning of the object relative clauses. Another subgroup showed severe difficulties both in reading the homographs in object relatives, and in paraphrasing the object relatives. The third subgroup showed relatively normal reading and paraphrasing of relative clauses. For this analysis, we classified each participant in the group of children with hearing impairment into the subgroups according to whether s/he failed in reading and whether s/he failed in paraphrasing compared to the hearing control group.

We defined failure in reading or paraphrasing as performance that is significantly below that of the hearing children. These comparisons of the performance of each of the experimental participants with the performance of the normative hearing control group were done using Crawford and Howell's (1998) *t*-test (see also Crawford and Garthwaite, 2002), *p* < 0.05. This test is a modification to the independent samples *t*-test that can be used to compare an individual, treated as a sample of *N* = 1, with a sample, in a way that the single participant does not contribute to the estimate of the within-group variance. This analysis according to failure in reading and/or paraphrasing created three subgroups that were confirmed also with a discriminant analysis. The two discriminant functions, using prior probabilities, predicted correctly 91.7% of the classification.

As summarized in Table 3, 11 children read the homographs in the relative clauses well (not significantly different from the control participants), but paraphrased them incorrectly, and significantly worse than the controls (*p* < 0.05). Their good reading of the homographs placed after the trace in object relatives indicates that these children are able to construct the syntactic structure correctly and to postulate an empty category in the trace position, and therefore they assume that the homograph is a verb, and not the object of the embedded verb. However, their failure to paraphrase the object relatives indicates that although they assumed an empty category in the right place, they were unable to establish the chain between the trace and the moved NP and hence did not assign the moved NP the correct thematic role. The good structure building of these participants goes well with previous descriptions of the Wh-movement deficit in hearing impaired children resulting from a problem in the chain of movement rather than from a deficit in the syntactic structure. For example, in a previous study (Friedmann and Szterman, 2011), we showed that the hearing impaired children in their study produced embedded sentences very well, indicating that they were able to construct the syntactic tree up to its highest node (we will explain in detail about the syntactic tree below).

**Table 3:** **Individual profiles: number of homographs in object relatives read correctly and of object relatives paraphrased correctly out of 10 object relatives**.

**Participant**		**Syntactic impairment**	**Homograph reading**	**Paraphrase**
**GOOD READING, POOR PARAPHRASING**				
1	DOD	Movement impairment	10	3
2	SIG	Movement impairment	9	5
3	TOS	Movement impairment	8	3
4	YEO	Movement impairment	10	3
5	YAO	Movement impairment	10	6
6	OFC	Movement impairment	8	2
7	TOH	Movement impairment	9	6
8	YOD	Movement impairment	10	6
9	ODC	Movement impairment	8	6
10	CBN	Movement impairment	9	3
11	YIC	Movement impairment	10	6
**POOR READING, POOR PARAPHRASING**				
1	DOH	CP impairment	7	4
2	AVC	CP impairment	4	1
3	IVL	CP impairment	7	3
4	ORC	CP impairment	4	1
5	DAC	CP impairment	7	1
6	LIS	CP impairment	7	6
7	HIM	CP impairment	8	4
8	NAH	CP impairment	5	5
9	YIB	CP impairment	5	3
10	LIH	CP impairment	7	2
11	LER	CP impairment	7	4
12	RAR	CP impairment	4	3
13	ZIZ	CP impairment	2	1
14	ORS	CP impairment	3	1
15	MAK	CP impairment	3	1
**GOOD READING, GOOD PARAPHRASING**				
1	CEB		10	8
2	TBN		10	8
3	SAV		10	8
4	NEA		9	8
5	KEM		10	9
6	ROS		9	8
7	TAM		10	9
8	YIL		10	9
9	ROP		10	10
10	DAM		9	9
11	TAC		10	7
12	SAS		10	7
13	RON		9	7
14	EDY		10	10
15	LIW		9	7
16	LIA		8	7
17	KOZ		10	10
18	LIC		10	10
19	TOS		10	10
20	LIL		9	9
21	MAL		9	7
22	AIR		9	7
Control group: average mean (SD)			9.4 (0.7)	8.9 (1.1)

Other 15 children showed poor reading of the homographs and poor comprehension of the relative clauses with the homographs (significantly poorer than the control group). Their reading indicates that it was not only their ability to assign thematic roles to the moved NP that was impaired. They did not even know that there was a movement in the sentence, and failed in the construction of the syntactic structure and the trace. How can this deficit be characterized? We suggest that it could be a structural problem in constructing the syntactic tree up to its highest nodes.

**Figure 2:**
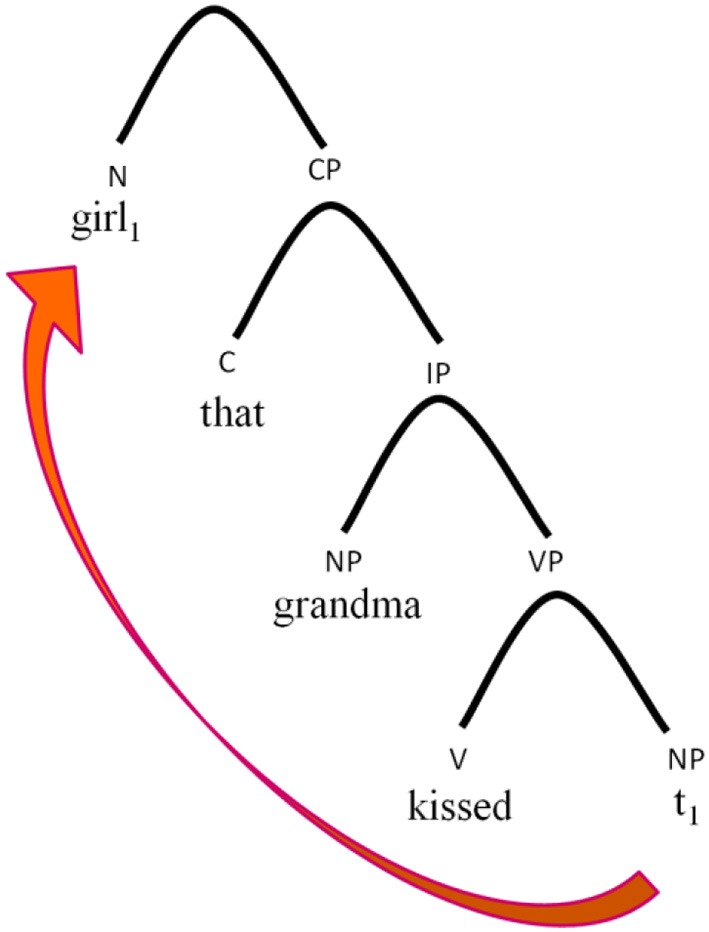
**The syntactic tree of a noun phrase with an object relative clause**.

When speakers produce or comprehend sentences, they represent them in syntactic trees (Pollock, 1989; Chomsky, 1995, see Figure 2). The phrasal architecture of the syntactic tree consists of three main structural layers, which are, from bottom to top, the lexical layer, the inflectional layer, and the complementizer layer (Chomsky, 1986, 1995; Rizzi, 1997). The lexical layer VP (verb phrase) contains the subject, the verb, and the object; the inflectional layer IP (inflectional phrase) is responsible for verb inflections; the CP (complementizer phrase) layer is responsible for embedding and for constituents that move to the beginning of the sentence such as Wh-morphemes and moving elements in relative clauses, verbs that move to second sentential position, and auxiliaries in yes/no questions in some languages. The CP-layer is the highest layer in the sentential hierarchy.

If these participants could not construct the tree up to the CP layer, which is responsible for embedding markers and Wh-movement, then they could not even know, when reading the sentence, that they need to be looking for a trace. Hence, they did not detect the trace position, which, in turn, led to their incorrect reading of the homograph. If this is a correct portrayal of their deficit, this has far-reaching predictions for the performance of this subgroup of hearing impaired children in other aspects of their linguistic performance—we would expect these children to show additional indications of CP impairment.

### Further assessment of CP in the subgroup with poor homograph reading

To determine whether the hearing impaired children who read the homographs incorrectly indeed have a structural deficit at the CP level, we looked at these children's pattern of errors in the current task, and also examined their performance in other sentence types and tasks that can serve as markers for the status of their CP. Their performance in both the current study and other tasks was revealing and supported the conjecture about a CP impairment.

Their reading of the relative clauses differed from that of the other hearing impaired participants not only in that they read the homograph as a noun. It also differed in another important aspect. When they read the sentences and reached the position of the embedding marker, they sometimes canceled the embedding [see example (14) above]. This problem with embedding was evinced both in their reading and in their paraphrasing of embedding. Thus, the reading pattern on the relative clause reading task, beyond the incorrect reading of the homograph, indicates a difficulty with embedding in this subgroup. This also affected the errors the children in this group made in paraphrasing the object relatives: whereas the children who were impaired in Wh-movement made mainly errors of thematic role assignment (79% of their errors) and the rest were interpreting the homograph as a noun (21%), the children with suspected CP impairment made many paraphrasing errors that stem from failed structure building: they had 54% errors of interpreting the homograph as a noun, 32% errors of thematic role assignment, and 15% paraphrases that disregarded the embedding. Importantly, each of the children in the CP group had errors of interpreting the homograph as a noun, and eight of the 15 children in the CP group showed cancelation of the embedding in their paraphrases. Thus, the children in the CP group made significantly more errors in paraphrasing that involved interpreting the homograph as a noun [*t*_(23)_ = 4.49, *p* = 0.0001], and canceling the embedding [*t*_(23)_ = 1.73, *p* = 0.048] than the children in the Wh-movement impairment group.

Findings from other tasks support the hypothesis regarding these children's inability to project the CP even in sentences that do not involve Wh-movement. We selected structures and tasks that are expected to be affected by a CP impairment, but not by a problem with the assignment of a thematic role to a moved NP that had undergone Wh-movement across another NP. We used tasks and structures that, as shown in Table 4, typically developing Hebrew-speaking children already perform very well in the age range of the hearing impaired participants. We had six tasks examining four different structures that corresponded to these criteria. One such structure are sentences with a subject relative clause, which include embedding but in which Wh-movement does not cross another NP. A deficit in the assignment of a thematic role to the moved NP across another NP would not affect the production of subject relatives, but a deficit in the CP layer is expected to affect subject relatives because they involve embedding and the CP layer. With a similar rationale in mind, we also looked at subject questions, which also involve the CP layer but no crossing movement. Subject questions, like subject relatives, are expected to be difficult if a child has a CP impairment but not if the impairment is constrained to Wh-movement across another NP. Two other structures with which we tested the CP layer were embedding without Wh-movement, with sentential complements of verbs (which involve the C node but no Wh movement), and sentences with verb movement to C, again, a structure that involves the CP layer but no movement of an NP across another NP.

**Table 4:**
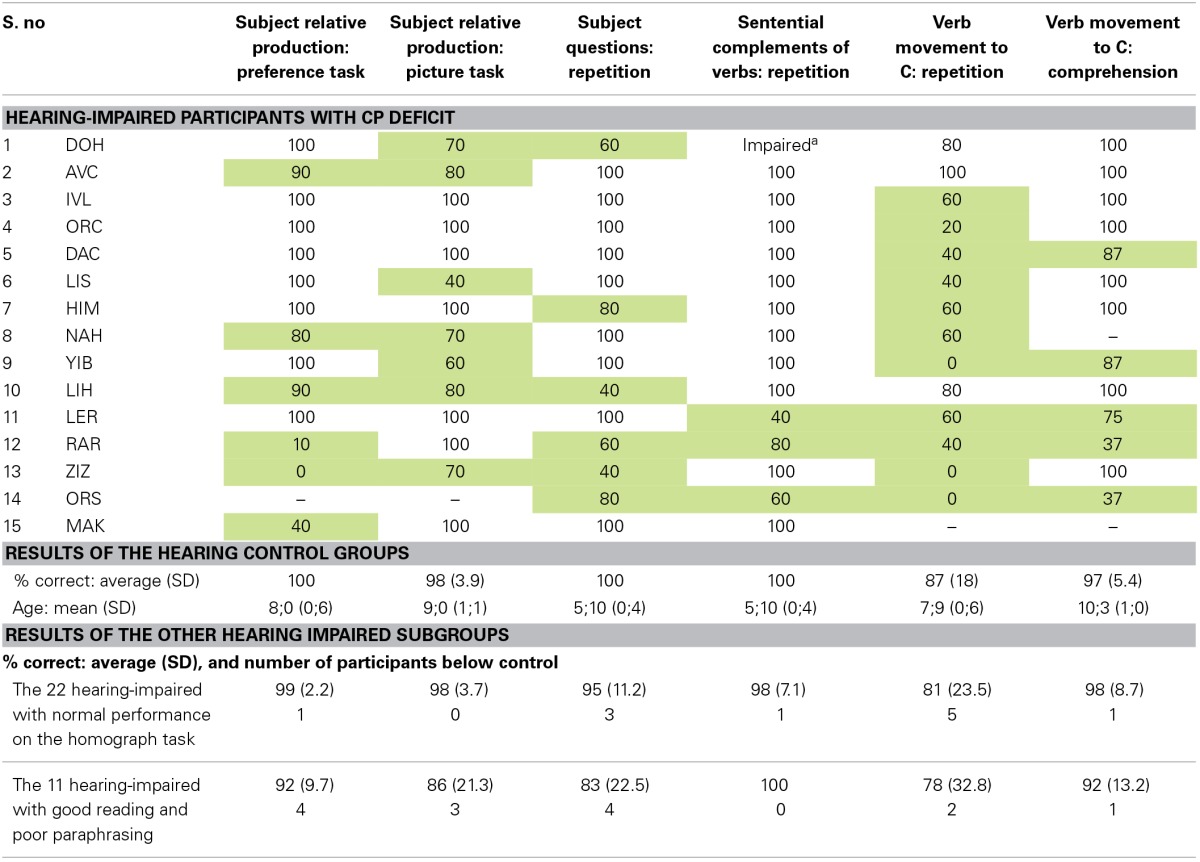
**Performance of the children with impaired homograph reading (and hence, suspected impairment in CP) in additional CP tasks, compared with hearing controls and the children with good homograph reading**.

*Shaded cells indicate that the participant performed this task significantly below the hearing control group*.

*^a^DOH repeated only three sentential complements, but a different task points to his embedding difficulty: in a test of pronoun comprehension, he performed only 50% correct in the embedded sentences (whereas in the simple sentences he performed well)*.

Because the comprehension of subject relatives and subject questions can rely on the canonical words order and possibly an agent-first strategy, we tested these structures using production and repetition tasks. Subject relative production was assessed using two tasks: a preference task in which the child heard descriptions of two children and was requested to choose the child he would rather be, using a subject relative clause (BAMBI ADIF, see Friedmann and Szterman, 2006; Novogrodsky and Friedmann, 2006, for details on this task), and a picture description task that elicited subject relatives (BAMBI ZIBUV, see Friedmann and Szterman, 2006, for details on this task. We used a repetition task (PETEL repetition task, Friedmann, 2000; see Fattal et al., 2011; Friedmann and Szterman, 2011 for details on this task) to evaluate the repetition of subject questions, as well as the repetition of sentences with a clause embedded to a verb and sentences with verb movement to C. The comprehension of sentences with verb movement to C was assessed using a task that is somewhat similar to the task described in the current article. The children heard sentences with the verb in second position after an adverb, in which a pseudoword was placed in the verb position or in the object position. Understanding whether the pseudoword is a noun or a verb required the correct construction of the sentence structure, including the verb movement to C. The children were then asked what the pseudoword could mean in each sentence, and we tested whether they suggested a verb or a noun (Szterman and Friedmann, 2011).

As shown in Table 4, each of the children who failed in the homograph reading task showed clear indications of difficulties in these CP-related tasks, and performed poorly in at least one task that the typically developing hearing children already perform very well in their age range or even in much younger ages^6^ ^,^^7^. Most of them (11 of the 15) failed on 2 or more such tasks. Importantly, the performance of the children with suspected CP impairment was not only much poorer than that of hearing children, but also poorer than the performance of the other subgroup of hearing impaired children, the ones who read the homograph correctly but failed to paraphrase the object relatives. As shown in Table 4, these children performed well on the CP tasks, much better than the hearing impaired children who failed in the homograph reading^8^.

Perusal of earlier literature that analyzed the performance of hearing impaired children in these tasks and structures sheds further light on the two profiles of impairment. First, it shows that other hearing impaired children who fail on Wh-movement can still perform well on tasks that involve CP but not Wh-movement. In previous studies Friedmann and Szterman (2011) assessed the comprehension and production of Wh questions and relative clauses in a different group of 18 Hebrew-speaking hearing impaired children. The production of subject and object questions was assessed using an elicitation task with 40 pictures of a figure doing something to another figure, in which the agent or the theme figure was concealed, and the child was instructed to ask a question about the concealed figure. Friedmann and Szterman found that 11 of the hearing impaired children failed to produce Wh questions in this task. Importantly, two profiles were detected in their production of Wh questions: Seven of the participants failed to produce object questions but still produced subject questions normally, whereas four participants showed difficulties in both object and subject questions. The difficulty in subject questions of these four children was also manifested in their poor comprehension and poor repetition of subject questions.

Similar results were found in a study that assessed the comprehension and production of relative clauses in Hebrew-speaking hearing impaired children (Friedmann and Szterman, 2006). Whereas 11 of the 14 participants performed better in subject relatives than in object relatives, 3 of the participants showed difficulties in both subject relatives and object relatives.

Finally, in a study of the comprehension of verb movement to C (Szterman and Friedmann, 2011), 9 of 12 hearing impaired participants performed well and similarly to the hearing control group (with no more than a single error) in the comprehension of sentences with verb movement. Three participants showed a different pattern, and failed to understand these sentences.

Thus, when previous data are analyzed at the individual participant level, they already suggest that two different patterns of syntactic impairments can be detected in hearing impaired individuals. We suggest that the 15 hearing impaired participants who failed to read the homograph in the object relatives in the current study had difficulty in the construction of the syntactic structure, presumably in the construction of the syntactic tree up to its highest node, CP. This difficulty was expressed in the way they read and paraphrased the object relatives, where they showed clear indications of difficulties in embedding, as well as in other tasks, in which they failed in the comprehension, repetition, and production of structures that did not involve Wh-movement (across another NP) but did involve CP: embedded sentences, verb movement to C, subject questions, and subject relatives. These structures are already mastered at this age by hearing children, and, importantly, by the hearing impaired children in the current study who read the homograph correctly also showed good performance in the CP tasks.

## Predictors of comprehension of movement-derived sentences

Given the two general patterns that we found: impaired and unimpaired syntax, and the further division into two profiles of impairment, we tried to see whether any of the background measures—depth of hearing loss, type of hearing aid, and age of fitting of a hearing aid—could be responsible for these groupings. We could not find any factor that determined, within the group of syntactically impaired children, who will show the movement deficit and who will show the CP deficit. However, the difference between the syntactically-impaired group and the group with normal syntax did correlate with one factor: whether or not hearing devices (be they cochlear implants or hearing aids) were fitted by the age of 1 year.

**The age of fitting of a hearing aid** was the only background factor that correlated with syntactic performance: Phi Coefficient of Association, calculated for the age of intervention (before or after 1 year) and performance (intact or impaired, determined using Crawford and Howell's, 1998, *t*-test as explained above), yielded a significant correlation, Phi = 0.44, *p* = 0.003, and the point biserial testing the correlation between paraphrasing of object relatives and whether or not hearing aids were fitted by 1 year of age also yielded a significant correlation, *r* = 0.42, *p* = 0.001. Namely, hearing devices (hearing aids or a cochlear implant) fitted by the age of 1 year gave the children a chance of having normal comprehension of relative clauses (as measured by normal paraphrasing of object relatives in our task).

In contrast, **depth of hearing loss** did not correlate with syntactic performance. There were children with profound hearing loss in the normal performance group (12 of the 22 children in this group), and there were children with only medium to severe loss in the syntactically-impaired subgroups (12 of the 26 children in these groups). A Point Biserial test for the correlation between the depth of hearing loss in dB (without hearing aid, measured in the better ear), and the performance of the participant in the object relative paraphrasing task (significantly below the control group or not) showed no significant relation, *r* = −0.05, *p* = 0.74.

The **type of hearing device** the child used also did not correlate with syntactic performance. There were 10 children with hearing aids and 12 with cochlear implants in the normal performance group, and 12 children with hearing aids and 13 with cochlear implants in the syntactically-impaired subgroups (9 with cochlear implants, 5 with hearing aids in the CP-impaired group, and 4 with cochlear implants, 7 with hearing aids in the Wh-movement impaired group). Phi Coefficient of Association that was calculated for the type of hearing aid (cochlear implant/hearing aid) and performance in the object relative paraphrasing (significantly or below the control group or not), also yielded no relation between the type of hearing aid and syntactic comprehension, Phi = −0.08, Fisher exact *p* = 0.77.

Finally, **Age** at the time of testing within the age group we tested also showed no correlation with performance: the three groups were of the same age ranges, and the point biserial correlation of age in month with performance was not significant: *r* = 0.12, *p* = 0.39.

## Discussion

The main questions of this study related to the nature of the syntactic deficit in hearing impairment, a question that took an interesting turn once we analyzed the individual profile of the participants rather than the group's, and to the relation between the syntactic impairment and reading in this population.

### On the relation between syntactic impairment and reading

This study examined various aspects of the relation between syntactic impairment and reading. The results indicated that hearing impaired children have difficulty understanding sentences derived by Wh-movement, and specifically, object relative clauses, not only when they hear these sentences, but also when the sentences are presented to them in writing, for an unlimited time. The ability of the group of hearing impaired children to interpret the written relative clauses, as reflected in their paraphrases, was significantly worse than that of hearing children. An individual level analysis indicated that 26 of the 48 hearing impaired participants performed significantly worse than the control group in interpreting the relative clause sentences.

This study thus sheds light on reading comprehension in the hearing impaired population. It indicates that individuals with hearing impairment have considerable difficulties in reading comprehension already at the sentence level, and this difficulty is clearly linked to their syntactic impairment, as their paraphrases of the simple control sentences were fine. Some of the hearing impaired participants even showed impaired reading aloud of object relatives, a difficulty that was linked to their syntactic impairment, rather than to a reading impairment. Again, this is supported by their good reading of matched simple sentences. These results open a window to the frequently reported difficulty of hearing impaired children in reading and in written text comprehension. They suggest that the text comprehension difficulties can result from a syntactic impairment.

Furthermore, this study showed that even problems in reading aloud of children with hearing impairment can be ascribed to their syntactic deficit, as the correct reading aloud sometimes requires the correct parsing of the syntactic structure of the sentence that they read. Thus, the syntactic impairment might cause not only difficulties in reading comprehension but also errors in reading decoding, depending on the syntactic structure of the target sentence.

### On the nature of the syntactic impairment in hearing impaired children

The major mission of this study was to explore the nature of the deficit in relative clauses in hearing impairment, and specifically to examine whether it is related to syntactic structure or to establishing a chain and assignment of thematic roles to a moved element.

In the test we used, the participants were asked to read aloud an object relative clause in which a homograph was placed after the gap position. This enabled us to see whether the participant was able to construct the syntactic structure of the relative clause and to postulate a trace in the required position. In general, the correct oral reading of heterophonic homographs depends on the semantic content of the sentence and on the correct analysis of its syntactic structure. Because we controlled for the semantic content in our sentences (both meanings of the homograph matched the semantics of the sentence), the oral reading of the homographs provided a sensitive marker for whether or not each participant was able to analyze the syntactic structure of the object relative correctly and to postulate the trace in the correct place.

One of the most striking findings of this study was that it exposed individual differences within the group of the hearing impaired participants who had difficulties in the comprehension of object relatives. Whereas some of them read the homograph correctly but failed to interpret the sentence, other participants could not even read the homograph. This principled variability could only be exposed when the individual performance was examined. It opens the window to important change in our understanding of the syntactic deficits underlying the comprehension and production difficulties in children with hearing impairment, as it exposes two different types of syntactic profiles.

The performance of the hearing-impaired participants who failed to understand the object relatives (namely, failed to paraphrase them) but read the homographs in the relative clauses correctly indicates that their syntactic structure was unimpaired. They could construct the syntactic tree including the CP node correctly, and could represent the embedding in the structure. Therefore, they could identify that there was a moved element (a filler) that they needed to find its original position (gap/trace). However, they could not link the original position (the gap) to the correct moved element and hence, could not reconstruct theta mapping and could not understand the role of the moved element in the embedded sentence. In processing terms (Nicol and Swinney, 1989), one may conceptualize their problem in the following way: they identified the gap position but could not re-activate the correct antecedent there. Therefore, as it were, they could not “undo” the movement operation (across an intervening NP), by reactivating the moved NP at the site of the gap. If one assumes that in online comprehension of an object relative the moved NP is kept in a syntactic STM store until it receives its thematic role, this difficulty can be a difficulty in selecting the NP to be reactivated between two similar NPs in the short-term syntactic store.

The children who failed to read the homograph, we suggest, had not identified the gap position from which the element has moved. Therefore these children not only fail to assign the thematic role to the moved element, they even cannot build the correct syntactic structure and do not assume the trace. We suggest that the deficit of this subgroup of hearing impaired children lies in the inability to construct the syntactic tree up to its highest nodes. Because they cannot construct the CP, the highest node of the tree, which hosts embedding and the moved element in object relatives, they do not know that they should expect a gap, and hence they completely fail to parse the sentence, and do not assume a trace. Support for their deficit in constructing the CP level came from their performance in the reading and paraphrasing task, as well from their performance in other tasks that involve the CP. In the reading and paraphrasing task these children often omitted and ignored the embedding marker or replaced it with another word. In the other tasks they showed, differently from the other hearing impaired children, a significant difficulty in the production of embedding markers, in elicited production and sentence repetition tasks. They also showed difficulty in the repetition of sentences with verb movement to CP, which the other hearing impaired children repeated correctly. Finally, they also showed a special pattern with respect to subject Wh-dependencies: Other hearing impaired children, such as the ones who read well but could not understand object relatives, typically find it difficult to produce object relatives and object questions, in which one NP is moved over another NP, but produce normally subject relatives and subject questions, in which the movement does not cross another NP. The 15 children with the CP deficit showed significant difficulty not only in the production of object relatives and object questions, but also in the production of subject relatives and subject questions. This indicates again that their difficulty was in the construction of the sentence with the CP node, which is required for both subject and object relative clauses and Wh questions (and not only in Wh questions in which one lexically-restricted DP crosses another).

Thus, we identify two profiles of syntactic deficit in children with hearing impairment: difficulty in creating the link between the moved NP and it original position (the trace), resulting in impaired understanding of the thematic role of the moved NP, and a deficit in building the syntactic structure up to its highest nodes. Researchers who tried to characterize the syntactic deficit of hearing impaired children suggested two different sources for the deficit: Friedmann and Szterman (2006, 2011) argue for a deficit in identifying the thematic roles in sentences in which a NP moved across another one, with good syntactic structure building. De Villiers et al. (1994), on the other hand, suggested that the deficit lies in constructing the high nodes of the syntactic tree. The current study shows that these researchers were both wrong and both right. It is incorrect that all hearing impaired children have intact syntactic structure (contrary to what Friedmann and Szterman, 2006, 2011 suggested), but it is also incorrect that all hearing-impaired children have a CP impairment (contrary to what De Villiers et al., 1994 proposed). Each of these characterizations, however, is correct about a different subgroup of hearing impaired children.

Looking at the background factors, one measure was clearly correlated with syntactic performance: the age at which hearing aids (or a cochlear implant) were fitted. Children who had their hearing devices up to 1 year of age showed significantly better syntactic ability than those who received hearing aids or a cochlear implant when they were older than 1 year. The type of hearing device (hearing aid or cochlear implant), depth of hearing loss (medium, severe, or deep), and age did not predict the syntactic performance. We could not find a background factor that determined, within the syntactically impaired children, who will show the movement deficit and who will show the CP deficit. The crucial effect that the age at which hearing aids are fitted has on later development of syntax points to the first year of life as a critical period for first language acquisition. Language input during the first year of life seems to be crucial for the development of normal syntactic abilities. This conclusion was also reached in earlier studies on children with hearing impairment (Szterman and Friedmann, 2003; Friedmann and Szterman, 2006). Other studies, which have tested language in general but not syntax specifically, also identified the age of identification of the hearing loss and age of initiation into intervention services as the most important predictor for normal language development (Apuzzo and Yoshinaga-Itano, 1995; Yoshinaga-Itano and Apuzzo, 1998a,b; Calderon and Naidu, 2000; Moeller, 2000; Mayberry et al., 2002; Mayberry and Lock, 2003; Yoshinaga-Itano, 2003). Evidence that further supports the importance of the first year of life in the normal development of syntactic abilities comes from a different population. Fattal et al. (2011, 2013) reported that children who did not receive thiamine, a vitamin necessary for brain development, during the first year of life showed severe syntactic difficulties when they were 5 and 9 year olds.

Returning to the two profiles of syntactic impairment, studies with other syntactically-impaired populations also show the two different sources for difficulties with relative clauses in two different populations. Children with Syntactic Specific Language Impairment (syntactic SLI) typically show a deficit that is best described as a deficit in Wh-movement, namely, in the assignment of thematic roles to an NP that moved across another NP (Friedmann and Novogrodsky, 2007, 2011; Friedmann et al., in press). Friedmann and Novogrodsky (2007) investigated this difficulty of Hebrew-speaking children with SySLI using the same task we used here. They found that the children with SySLI read the homographs well, but failed to paraphrase the object relatives. The performance in this task was interpreted as indicating a deficit in movement. Namely, the children with SLI could not activate the correct NP at the trace position. When this happened, the SySLI participants failed to assign the correct thematic role to the moved element, and this led to various paraphrases in which the thematic roles were incorrectly assigned to the arguments. This interpretation is supported by a study by Marinis and van der Lely (2004), who used cross-modal lexical priming and found that English-speaking children with SLI were unable to reactivate the antecedent at the Wh-trace position. That is, even if they know where to place an empty category, they do not know to which phrase to link it, and hence they cannot assign the thematic role correctly.

Another population with a syntactic impairment is that of individuals with agrammatic aphasia. Individuals with Broca's agrammatic aphasia show significant difficulties in the comprehension of sentences derived by syntactic movement that result in a non-canonical order of the arguments such as object relative clauses, object Wh questions, and topicalization structures (Zurif and Caramazza, 1976; Schwartz et al., 1987; Grodzinsky, 1989, 2000; Grodzinsky et al., 1999; Friedmann and Shapiro, 2003). Friedmann et al. (2006) explored the nature of this deficit using the test we used in the current study (a longer version of it), with individuals with agrammatism. They found that all the individuals with agrammatism they tested were severely impaired in reading the homographs when they appeared after the trace in the object relative clauses, and consequently failed to paraphrase them. Friedmann et al. (2006) interpreted this pattern as evidence for a difficulty in constructing the CP node, a deficit that has abundant evidence for from other sentence production tasks (Friedmann, 2001, 2006). Thus, it seems that some of the hearing impaired children who show a deficit in the comprehension of object relatives have a deficit that is similar to that of children with syntactic SLI, whereas others show a deficit that resembles that of individuals with agrammatism.

When the results regarding the deficit in comprehension and the two different profiles of impairment are brought together with the impression and report of the clinicians and special education teachers who work with these children, some important clinical implications emerge. Whereas all the clinicians and teachers of the hearing impaired children easily detect the syntactic difficulties of the children who had the syntactic structure deficit, and these children are classified with “severe language impairment”, the syntactic difficulties of the children who read the sentences correctly is more elusive. Because these children can produce embedded sentences well, and can even read sentences correctly, it is harder to suspect that they have a syntactic deficit. It is thus crucial to test the comprehension of semantically reversible sentences derived by movement even for hearing impaired children who do not display an obvious syntactic deficit in their speech and reading aloud.

One final point relates to whether the deficit of the hearing impaired children who showed impaired comprehension in this task indeed related to Wh-movement or rather to center embedding, as all the object relatives in this study were center-embedded. The participants in this study were part of a wide study in which they were also tested for the comprehension of other Wh-movement structures, including Wh questions, topicalized OSV sentences, and final-branching object relatives in sentence-picture matching tasks and in questions on written sentences. The results of these other tasks indicated that each of the participants who failed in the paraphrasing of the object relatives in the reading task also showed difficulties in the comprehension of object Wh-movement without center embedding (significantly poorer performance in the comprehension of at least one of the above structures). This indicates that they had a genuine deficit in Wh-movement rather than a deficit related to center embedding.

This study thus shows not only that hearing impaired children have syntactic difficulties, but also that these difficulties can have different faces. These difficulties can result from an impairment in syntactic structure building or from an impairment in chain formation: a deficit in linking a moved NP to its original position and hence, impaired assignment of thematic role assignment to the moved NP. The study further suggests that the reading comprehension and reading aloud difficulties can be tightly related to syntactic difficulties, and shows the importance of paying attention to variability within language-impaired groups (see also Coltheart, in press), by analysis of individual performance.

### Conflict of interest statement

The authors declare that the research was conducted in the absence of any commercial or financial relationships that could be construed as a potential conflict of interest.

## Footnotes

^1^Some analyses assume that relative clauses are constructed by a movement of the head NP from inside the embedded relative clause CP to the relative head position above CP (raising analysis). Other analyses (matching analyses) suggest that it is an empty operator that moves from within the embedded sentence. It moves to the specifier position of CP, where it is co-indexed with the head of the relative clause (see Vergnaud, 1974; Chomsky, 1986, 1995; Kayne, 1994; and see Sauerland, 2000, for a discussion of the two analyses). For the purpose of the current study, the differences between these two approaches are irrelevant.

^2^The hyphens between two morphemes or two words in the Hebrew examples indicate that they form a single written word in Hebrew.

^3^ Using a definition of ambiguity bias according to which the difference between the number of judges who preferred the noun meaning and the number of judges who preferred the verb meaning was at least 25% (13) of the judges there were six words biased toward the (incorrect) noun reading, and four which were unbiased.

^4^In Hebrew, compounds like “tower of curls” are two-word phrases without the word “of” (Doron and Meir, 2013).

^5^Another finding that supports the idea that impaired reading-decoding abilities do not underlie the hearing-impaired participants' failure on object relatives in this task is that individuals with developmental dyslexia (surface dyslexia, letter position dyslexia, attentional dyslexia, and visual dyslexia) without a syntactic problem who were tested on the same task still read the homograph in the relative clauses well, and similarly to their reading of the same homograph in the simple control sentences, and could interpret the relative clauses correctly (Kesselman et al., 2013).

^6^ The results of the hearing children in Table 4 are taken from previously reported data that used the same tests. The data on subject relative clause elicitation is from Fattal et al. (2013) for the preference task and from Novogrodsky and Friedmann (2006) for the picture task, the data on the repetition of subject questions and sentences with sentential embedding are taken from Fattal et al. (2011), the data on the repetition of sentences with verb movement to C are taken from Fattal et al. (2013) and the data on the comprehension of sentences with verb movement to C is taken from Szterman and Friedmann (2011).

^7^The poor performance on the comprehension and repetition task could not be ascribed to the participants' not hearing the sentences well. These tasks included simple control sentences on which the participants performed well, indicating that hearing the sentences was not the problem, but rather their syntactic structure. Recall, also, that all the participants passed a hearing screening test (see Participants section).

^8^ One interesting point relates to the performance of the subgroup with the Wh-movement deficit in subject Wh-movement. The table reveals that a few of the Wh-movement impaired individuals also showed (slight) difficulties in production even of subject dependencies. This opens a further question of whether the movement-impairment variety of syntactic impairment can also be sub-divided into children with a deficit only in wh-dependencies in which one lexically-restricted NP crosses another, and children who have a problem with any type of Wh-movement. (These children may still show good performance in comprehension tasks such as sentence-picture matching with subject questions and subject relatives, where they may use an agent-first strategy, a strategy that cannot be employed in production.)
